# Immune responses in the uterine mucosa: clues for vaccine development in pigs

**DOI:** 10.3389/fimmu.2023.1171212

**Published:** 2023-07-07

**Authors:** Pooja Choudhary, Donaldson Magloire, Glenn Hamonic, Heather L. Wilson

**Affiliations:** ^1^ Vaccine and Infectious Disease Organization (VIDO), University of Saskatchewan, Saskatoon, SK, Canada; ^2^ Department of Veterinary Microbiology Sciences, Western College of Veterinary Medicine, University of Saskatchewan, Saskatoon, SK, Canada

**Keywords:** uterus, vaccine, adjuvants, fertility, pigs, livestock, intrauterine

## Abstract

The immune system in the upper reproductive tract (URT) protects against sexually transmitted pathogens, while at the same time providing immune tolerance responses against allogenic sperm and the developing fetus. The uterine environment is also responsive to hormonal variations during the estrus cycle, although the most likely timing of exposure to pathogens is during estrus and breeding when the cervix is semi-permissive. The goal for intrauterine immunization would be to induce local or systemic immunity and/or to promote colostral/lactogenic immunity that will passively protect suckling offspring. The developing fetus is not the vaccine target. This minireview article focuses on the immune response induced in the pig uterus (uterine body and uterine horns) with some comparative references to other livestock species, mice, and humans.

## 1 Introduction

Unlike many other large livestock animals, pigs are not immobilized in headgates during handling; therefore, delivery of vaccines can be difficult and a potential safety hazard, especially in terms of the risk of needle-stick injuries (1). Before immunization can take place, the animals need to be snared or moved into crates, both of which require more than one person. Like other agriculture sectors, the pig industry is experiencing a labor shortage, and novel ideas to eradicate inefficiencies within the barns may help bridge these gaps. To that end, vaccines that can be administered without needles and at a time when animals are immobilized (without the need for multiple workers) would be well-received by the swine industry. Sows and gilts are bred during estrus when the cervix is semi-permissive and, upon being exposed to the boar or boar pheromones, they become temporarily rigid in preparation for mounting, referred to as lordosis (2). Because they are temporarily immobilized, the sows and gilts are safe to immunize and other barn personnel are not required to snare the animals, meaning that two tasks can be accomplished at once (breeding and immunization) with reduced laborers. Further, because breeding takes place more than two times per year, the timing of intrauterine immunization is optimal for induction of immunity (3–5). Reports on the innate and adaptive immune response in the uterus support the hypothesis that the uterus may be amenable to an immune induction site, although care should be taken to avoid induction of non-tolerizing response to sperm and seminal fluid constituents.

## 2 Innate immune responses in the uterus and uterine horns

It has lately been appreciated that the uterus and uterine horns have their own microbiome (6). The gut microbiome in the sow can be modulated in response to antimicrobials, reproductive stages, feed and supplements, pathogen exposure as well as vaccines (reviewed in (7)). The impact of these factors on the sow uterine microbiome needs to be explored, as does the potential impact that an intrauterine vaccine may have on the microbiome of the uterus and other sites.

The uterine microenvironment is under immune surveillance and is reactive to foreign antigens (8). Sperm deposited in the uterus triggers a natural immune response, which serves both to clear the uterus of excess sperm (9, 10) as well to accommodate the embryo for implantation (11). Studies in livestock bred by AI showed that spermatozoa, seminal plasma, and extender trigger rapid and transient neutrophil infiltration into the lumen and an inflammatory response, complete with cytokine and chemokine induction (12–15). Studies in pigs show that semen extender and seminal plasma alone induced interleukin (IL)-10, transforming growth factor (TGF)-β, IL-8, and tumor necrosis factor (TNF)-α gene expression but that when combined with spermatozoa, the expression of these genes is reduced (16). Sperm has been shown to promote immune tolerance via signaling through Toll-like receptor (TLR) 4 (17, 18). Furthermore, seminal fluid antigens activate regulatory T cells in the uterus draining lymph node, which dampens the immune response in the uterus to tolerate the antigens present in the embryo (19).. Others show that semen extender triggers induction of granulocyte-macrophage colony-stimulating factor (GM-CSF) and a corresponding increase in major histocompatibility complex (MHC) class II-positive cells in the uterine lamina propria and directly basolateral to the epithelial layer (20). The extent to which a vaccine coupled with artificial insemination will influence the sperm and seminal fluid’s role in evoking or dampening an immune response has not been fully elucidated.

Recruitment of antigen-presenting cells (APC) to the lumen or the uterine tissue may be key to an effective intrauterine (i.u.) vaccine that is delivered in combination with a semen extender. Boar semen combined with three adjuvants (poly I:C, host defense peptide, and polyphosphazene (Triple Adjuvant; TriAdj)) administered to the uterus was shown to trigger changes in localized gene expression and cellular recruitment *in vivo* and greatly increased the number of neutrophils in the uterine lumen (12, 21). These data suggest that sperm, semen, semen extender, and adjuvants may augment local immune response and, therefore, may influence the immune response to intrauterine vaccines. Our results in pigs have shown that vaccines coupled with sperm at the time of breeding does not affect reproduction, but the effect of multiple breedings/vaccinations should investigate whether anti-sperm antibodies do increase (22). Caution should be used to formulate the vaccine so that it does not trigger the induction of anti-sperm antibodies, which may cause infertility (23).

## 3 The uterus as an immune induction site

Intrauterine immunization is a novel approach and currently, published data have been limited to rodents and pigs. In mice, prior i.u. exposure to live *Chlamydia trachomatis* (Ct) generated protective immunity against subsequent challenge, suggesting that the uterus can act as an immune induction site (24, 25). Further, immunization with ultraviolet light (UV)-inactivated Ct (UV-Ct) complexed with charge-switching synthetic adjuvant biodegradable nanoparticles (cSAPs) elicited long-lived protection in conventional and humanized mice (24). Mice immunized with UV-Ct alone generated regulatory T cells and an accumulation of tolerogenic CD11b^-^CD103^+^ dendritic cells (DCs) that exacerbated subsequent Ct infection, whereas mice immunized with UV-Ct-cSAP exhibited elevated immunogenic uterine CD11b^+^CD103^-^ DCs that led to effector T cells seeding the uterine mucosa with resident memory T (T_RM_) cells (24). These data suggest that the inclusion of mucosal adjuvants may be critically required for protective i.u. immunization.

The hormonal state may also impact the immune response as although DCs in the decidua of pregnant mice retain responsiveness to pro-inflammatory stimuli and migration capacity towards CCL21, these cells are prevented from trafficking to the draining lymph node (LN), possibly to promote T cell tolerance to fetal antigens (26). These data suggest that DCs may have region-specific or timing/hormone-specific responses. Understanding the mechanism of action of adjuvants and the cells they target should continue to be a research focus for i.u. vaccines.

Recent studies in pigs show that the uterus can act as a site of booster immunization and/or as an immune induction site. In a previous study, sows that had been immunized repeatedly with the Porcine ParvoShield L5E Swine Vaccine^®^ (Elanco Animal Health) by the intramuscular (i.m.) route at each parity were bred with semen alone or semen plus BEI-inactivated porcine parvovirus (1 × 10^7^ TCID_50_ PPV (NADL-7) formulated with a combination adjuvant (TriAdj; host defense peptide, poly I:C, and polyphosphazene) (21). This vaccine was not spermicidal. Positive control sows received i.m. ParvoShield^®^ vaccine as they entered farrowing crates. Serum antibody titres against viral protein 2 (VP2, one of the capsid proteins of PPV) were comparable between the positive control sows and sows immunized by the uterine route, suggesting that the uterus could act as a site of booster immunization to an inactivated virus (21). When sows were bred with the immunogenic recombinant spike protein from a porcine epidemic diarrheal virus (rPEDVS) plus TriAdj adjuvants, the anti-PEDVS serum and uterine antibodies were low in the i.u.-vaccinated gilts (22). These data suggest that a single primary immunization delivered into the uterus may not be sufficient to evoke a systemic or mucosal humoral immune response (21). Importantly, there was no difference in the viable fetus/corpus luteum ratio after 30 days between i.u.-vaccinated and control sows, suggesting that the i.u. vaccines did not impact fetal development.

To assess whether multiple i.u. vaccines could trigger a robust immune response, gilts were bred with heat-inactivated extended semen containing rPEDVS formulated with TriAdj. The gilts returned to estrus after 21 days and they were rebred with the same inactivated semen and vaccine, suggesting that i.u. immunization did not impact hormonal cycling. When they returned to estrus again, they were bred with live semen plus the vaccine (22). Control gilts were administered semen alone at second estrus following common industrial breeding practices. Litter weights and the number of live to non-viable piglets were comparable, indicating that the three-times-administered i.u.-vaccine did not appear to impact fertility. The i.u.-vaccinated gilts showed significant PEDVS-specific serum, colostral, and uterine antibody titers, and low-level colostral PEDVS-neutralizing antibodies. Serum from piglets born from i.u immunized gilts showed increased antibody titers compared to control piglets (22), which showed that i.u vaccines can induce higher maternal antibodies. Piglets born to i.u.-vaccinated gilts received partial passive protection from PEDV infection 3 days after birth but eventually succumbed to the disease (22). Collectively, these data indicate that the porcine uterus could act as an immune induction site, but that more than one dose is needed, at least when TriAdj is used as the vaccine adjuvant (22). In a follow-up trial, the rPEDVS vaccine was formulated with polymeric poly-(lactide-co-glycolide) (PGLA)-nanoparticle (NP) including a muramyl dipeptide analog and a monophosphoryl lipid A (MPLA) analog as adjuvants (NP-PEDVS) (27). The gilts responded with significant induction of serum anti-PEDVS-IgG following the single dose after 30 days, suggesting that an NP vaccine may be suitable for primary i.u. vaccination. Collectively, these experiments indicate that the pig uterus can act as an immune inductive site when the vaccine is administered at breeding, but that the use of robust adjuvants (that are formulated to not be spermicidal) may be critical to vaccine efficacy. In addition to rodents and pigs, it would be interesting to see the results on other animals that breed via AI such as cattle and horses.

Intradermal (i.d.) vaccination is also an alternative to i.m. immunization which results have shown can induce a comparable or better immune response (28). In some instances, i.d. immunization requires needles administered using the Mantoux technique, which requires 5–15° injection angle into the skin going approx. 1 mm deep (29–31). This technique requires persons be trained and it can be difficult to use on a non-anesthetized animal (31, 32). Administration of i.d. vaccines using bifurcated needles or multipuncture devices can be complicated by uneven antigen delivery (33). In contrast, i.u. immunization takes advantage of breeding practices and does not require specialized skills from the administrator.

## 4 Mechanism of action

### 4.1 Uterine epithelial cell pattern recognition receptors

As in other mammals, the porcine uterus/uterine horn is lined with a single layer of simple columnar cells with tight junctions between the cells to control passage, and the tight junctions are regulated by the hormonal state, cytokines, growth factors, TLR agonists, and pathogens (34–36). The underlying endometrial layer has a superficial functional layer (*stratum functionale*) and a deeper basal layer (*stratum basale*) with glandular epithelial cells forming tubular glands that spiral into the tissue (37, 38). The endometrium undergoes changes in the branching of the glands and growth, including changes in endometrial thickness and epithelial cell height in response to the estrus cycle (37, 39, 40).

The epithelial and endometrial layer of the uterus expresses several pattern recognition receptors that may play a role in the uterine immune response. TLRs are membrane-spanning receptors on the uterine epithelial cell surface that identify the pathogen-associated molecular patterns (PAMPs) present in bacterial, fungal, and viral pathogens and initiate innate immune responses (41, 42). Analysis of mRNA expression levels performed on isolated and cultured pig primary uterine epithelial cells showed that TLRs 1-7 and 9, NOD1, NOD2, NLRP3, NLRP6, NLRX1, RIG1, MDA5, and LGP2 are expressed (43). Polarized primary uterine epithelial cells (UECs) stimulated with TLR3, TLR4, and TLR9 ligands showed induced secretion of IL-6, IL-13, and IL-10, respectively, indicating that these receptors were functional (43). Polarized uterine epithelial cells stimulated with a TLR3 agonist showed increased expression of interferon (IFN)-β, TNF-α, IL-8, CCL2, CCL3, CCL4, and CCL-20 (21). Further, laser-captured uterine epithelial cells obtained one day after being bred with semen plus an adjuvant cocktail containing a TLR3 agonist showed significantly increased CCL2, suggesting that pig uterine epithelial cells are responsive to immune stimuli (21). Immunohistofluorescence and immunofluorescence performed on pig uterine tissue and in polarized pig primary UECs indicated that TLR3 and TLR9 localizes to the apical cell surface, whereas TLR4 localizes to the intracellular space (43). Surface localization of TLR3 in pig uterine epithelial cells shows agreement with uterine epithelial cells in humans (44, 45) and rabbits (46); continued research in this area shows that the ‘canonical’ localization patterns of TLRs may not be conserved across the cell and/or tissue types and may also vary in response to stimulation, age, disease, or cellular environment (reviewed in (43)).

### 4.2 Immune cells in the uterine lumen and endothelium

Cells in the uterine lumen and endometrium are sensitive to changes in the hormonal environment. We limited the scope of this mini-review to the estrus cycle when the cervix may be permissive to vaccines. Immune cells in the endometrium are primarily lymphocytes, with some macrophages and APCs and large numbers of neutrophils; these levels tend to be at their highest during estrus (38, 40). Macrophages, DCs, lymphocytes, and granulocytes migrate from the blood to subepithelial tissue where they may persist (20, 47). Neutrophils are found close to the basal lamina of the surface epithelium and the subepithelial capillaries at pre-estrus and estrus (48, 49). They migrate into the uterine lumen after breeding where they eliminate a large number of spermatozoa and microbes present in boar semen (47, 50) and they usually die after 24 hours (20).

Uterine APCs, such as macrophages and DCs, are present throughout the endometrium during estrus; however, at other stages of the estrus cycle, they are found deeper in the lamina propria and rarely reside directly below the surface epithelium (40, 51). In mice, macrophages can be identified by the expression of the cell surface F4/80 and can be positive or negative for the surface marker CD11c (52, 53).. Macrophages are the most abundant professional APC in the human uterus (54). DCS can be characterized into 3 major subsets: plasmacytoid DC (pDC), conventional DC1 (cDC1), and conventional DC2 (cDC2) populations based on cell-surface markers and transcription factors such as interferon regulatory factors 8 and 4 (IRF8 and IRF4) (55–57). Once DCs capture antigens, they become mature and migrate to lymphoid structures to present antigens to T cells (58). DCs in the uterus are characterized as having high levels of major histocompatibility complex class II. In mice, cDC1 cells in nonlymphoid tissue, such as the uterine tissue, can be either CD103^+^CD11b^−^ cDC1 and CD11b^+^ cDC2 (59). CD103^+^ cDC1 has two principal functions, i.e., priming CD8^+^ cells by cross presentation and induction of tolerance (60). pDC1 cells mainly produce high type I IFN in response to viral infection (61) whereas CD11b^+^ cDC2 drives the CD4^+^ T helper 2 (TH2) and 17 (TH17) response (62). In pigs, subsets of DCs are identified and characterized as cDC1: CD135^+^CD14^-^CD172a^low^CADM1^+^wCD11R1^+^ cells; cDC2: CD135^+^CD14^-^CD172a^+^CADM1^+^CD115^+^wCD11R1^+^CD1^+^ cells; and pDCs: CD4^+^CD135^+^CD172a^+^CD123^+^CD303^+^ (63).

In pigs, plasma cells are dispersed throughout the endometrium with a predominance of IgG-secreting plasma cells (38, 38). The most prevalent cell type at all stages of the estrus cycle is the CD2^+^ cell (48) with CD8^+^ cells being present more frequently than CD4^+^ T cells in the surface epithelium compared to the CD4^+^, and more CD4^+^ cells than CD8^+^ cells in the glandular connective tissue (51). In the connective tissue of the subepithelial layer, there is no significant effect of the estrus cycle stage on the numbers of CD2^+^, CD4^+^, and CD8^+^ cells (49).

### 4.3 Antigen presentation

For an immune response to occur, APCs need to internalize the antigen, process it, and present it to T cells on MHCI or MHCII proteins (64, 65). The quality and direction of the adaptive response depends on how the APCs react to the adjuvant, leading to the secretion of select cytokines (66, 67).. In the human uterus, antigen presentation on MHC class II can be performed by professional APCs as well as uterine epithelial cells (68, 69). In contrast, in pigs, SLA-DRA gene expression was not detected in any uterine epithelial cells, indicating that pigs do not express the porcine equivalent of MHC class II (21). These data suggest that there are species-specific differences between the roles of epithelial cells in immune activation in the uterus and that pig uterine epithelial cells do not act as APCs.

The upper reproductive tract does not contain the mucosal-associated lymphoid tissue (MALT) thought to be critically required for the induction of an immune response (70). There is limited evidence that the pig’s upper reproductive tract has lymphoid aggregates that may be acting as a limited MALT. One study showed that in 3 out of 6 sows studied, aggregations of lymphocytes were noted in the subepithelial connective tissue of the cervix (https://stud.epsilon.slu.se/12300/1/edstrom_k_171031.pdf). Another study referred to an unpublished observation that reported 50% of ancestral multiparous sows had a few small lymphocyte aggregations in the endometrium at weaning (71). Other studies show that lymphoid aggregates are present throughout the uterus, usually within the surface and in the glandular epithelium, and vary in size throughout the estrous cycle stages (72, 73). In the glandular epithelium, CD4^+^ cells are absent, CD8^+^ cells typically increase during estrus whereas CD2^+^ cells are at most during estrus and early diestrus. On the surface epithelium, more CD4^+^ are found over CD8^+^ cells during estrous, whilst CD2^+^ cells are in high numbers both during estrus and early diestrus (73).

The human uterus has lymphoid aggregates in the endometrial tissue at the basal and functional area of the uterus near the uterine epithelial glands (74). These aggregates are comprised of CD19^+^ B cells surrounded by numerous (and primarily CD8^+^) T cells and an outer layer of monocytes or macrophages (75, 76). The size of the lymphoid aggregates varies with the estrus cycle. It appears to be the largest during the secretory and proliferative phases; this is consistent with an increase in the immune cell trafficking into the endometrium, which may contribute to the larger lymphoid aggregate in the secretory phase (75, 77).

In the subepithelial stromal layer of the endometrium of the bovine uterus, isolated lymphoid nodules and aggregates predominantly comprised of clusters of B and T-lymphocytes have been reported (78, 79) (80). B lymphocytes were also observed as a small aggregate deep in the stroma or adjacent to blood vessels in the myometrial layer of the uterus, possibly recruited to the mucosal surfaces in response to the chemokines secreted during infection (81–83). These studies indicate there may be species-specific differences in how vaccines in the uterine lumen mediate the immune response, and/or it is possible that uterine vaccination would require transport of the vaccine across the epithelial barrier.

### 4.4 Transport of molecules across the epithelial barrier

Tight junctions between UECs limit the transport of molecules across the epithelial barrier. The predominant method for transporting macromolecules across an epithelial cell wall would be through pinocytosis, which involves transporting macromolecules into or across the cell using vesicles. Pinocytosis is a form of endocytosis that is not receptor-mediated and is therefore non-specific. Pinocytosis involves the internalization of the plasma membrane to form a vesicle that contains extracellular fluid, and any molecules present in that fluid. Studies in other epithelial cell barriers such as in alveoli and the intestine have shown that pinocytosis occurs in a non-specific fashion and transports macromolecules across the epithelial cells at a rate proportional to their size (84) and that negatively charged nanoparticles are more efficiently transported (85). Although there is limited data on the mechanisms of pinocytosis by UECs and how the size or charge of particles impacts their transport, there is evidence of molecules being transported in a luminal to basolateral direction, which could be used by intrauterine vaccines (86).

Another mechanism of transport across the uterine epithelial cell barrier may include receptor-mediated transport using antibody transporters. For example, despite its name, neonatal Fc receptor (FcRN) is expressed by both porcine and human UECs into adulthood (87, 88). IgG can be bi-directionally transported between neutral environments through FcRN-mediated transport (89, 90). The transfer of IgG by FcRN in the human female reproductive tract has been confirmed (87). It is possible that by binding to the vaccine antigen, FcRN-IgG transportation could deliver the antigen and possibly the associated vaccine components across the epithelial wall. Further, polymeric immunoglobulin receptor (pIgR)-mediated transport for the secretion of sIgA has been well described and is known to be carried out by uterine epithelial cells (88), although IgA is not the predominant immunoglobulin secreted into the uterine lumen. Thus, antibodies bound by these receptors could transport coupled antigens across the epithelial barrier. Future studies should investigate where the vaccine components localize and are taken up by innate immune cells when they are formulated as soluble or particulate vaccines.

A summary of how intrauterine immunization can impact the pig industry and factors that may influence immune activation are presented in **Table 1**.

**Table 1:** Impact of intrauterine immunization.

Impact on industry		Factors that may influence immune activation
Positive attributes	Potential challenges	
•Needle-free •Safe to immunize during lordosis response •Coupling breeding with immunization reduces labor requirements	•Assess long-term impact on fertility •Must ensure sperm are not targeted and no negative effect on microbiome	•Uterine epithelial cell pattern recognition receptors •Hormonal changes in transport receptors, cell numbers, and cell localization •Site of immune activation is not yet clear. •Uterus has limited lymphoid aggregates instead of MALT •UECs do not present antigens to T cells

## 5 Discussion

The uterus is known to exhibit inflammatory responses and there is evidence that it can act as an immune induction site. Studies are needed to determine whether intrauterine vaccines trigger immune cell recruitment into the lumen, which is critical for induction of immunity, or whether the antigen traverses the uterine wall to trigger immunity. How the antigen traverses the uterine wall (i.e., via paracellular transport or transcytosis, uptake by dendritic cells extending dendrites into the lumen, etc.) and whether the antigen is presented to draining LNs or lymphoid aggregates should also be investigated. Once it is clear how the uterus acts as an immune induction site, vaccines can be formulated to exploit this mechanism of action. Coupling breeding with vaccination should reduce the number of personnel required for handling and would not require any special training, making it a potentially important new route of immunization for the pig industry. The effect of multiple rounds of i.u. immunization on sperm tolerance and the uterine microbiome must be investigated further, with each new vaccine formulation.

## Author contributions

PC, DM, and HW researched and wrote the initial draft. All authors contributed to revising and reviewing the manuscript. Portions of this manuscript are also found in the Ph.D. thesis for GH, used with permission. All authors contributed to the article and approved the submitted version.

## References

[1] Hafer AL Langley RL Morrow WEM. Occupational hazards reported by swine veterinarians in the united states. J Swine Health Production (1996) 4(3):128–41.

[2] Dorries KM Adkins-Regan E Halpern BP. Sensitivity and behavioral responses to the pheromone androstenone are not mediated by the vomeronasal organ in domestic pigs. Brain Behav Evol (1997) 49(1):53–62. doi: 10.1159/000112981 8980852

[3] McNamara KA Sloter N L Zas Rodriguez S L Knox R V Gall T J Levis D G. An analysis of survey data by size of the breeding herd for the reproductive management practices of north American sow farms. J Anim Sci (2013) 91(1):433–45. 10.2527/jas.2012-5189 23097401

[4] Knox RV. Artificial insemination in pigs today. Theriogenology (2016) 85(1):83–93. doi: 10.1016/j.theriogenology.2015.07.009 26253434

[5] Hernández-Caravaca I Izquierdo-Rico MJ Matás C Carvajal JA Vieira L Abril D. Reproductive performance and backflow study in cervical and post-cervical artificial insemination in sows. Anim Reprod Sci (2012) 136(1):14–22. doi: 10.1016/j.anireprosci.2012.10.007 23141953

[6] Rampersaud R Randis TM Ratner AJ. Microbiota of the upper and lower genital tract. Semin Fetal Neonatal Med (2012) 17(1):51–7. doi: 10.1016/j.siny.2011.08.006 PMC3242913 21920833

[7] Monteiro MS Poor AP Muro BBD Carnevale RF Leal DF Garbossa CAP. The sow microbiome: current and future perspectives to maximize the productivity in swine herds. J Swine Health Production (2022) 30(4):238–250. doi: 10.54846/jshap/1277

[8] Robertson SA. Control of the immunological environment of the uterus. Rev Reprod (2000) 5(3):164–74. doi: 10.1530/ror.0.0050164 11006166

[9] Zambrano F Poor AP Muro BB.D Carnevale RF Leal DF Garbossa CA.P. Leukocytes coincubated with human sperm trigger classic neutrophil extracellular traps formation, reducing sperm motility. Fertil Steril (2016) 106(5):1053–1060.e1. doi: 10.1016/j.fertnstert.2016.06.005 27344301

[10] Hong J Dicker BL Jayasinghe SN Gregorio De F Tian H Han DY. Strong inhibition of neutrophil-sperm interaction in cattle by selective phosphatidylinositol 3-kinase inhibitors. Biol Reprod (2017) 97(5):671–87. doi: 10.1093/biolre/iox121 29036279

[11] Troedsson MH Loset K Alghamdi AM Dahms B Crabo BG. Interaction between equine semen and the endometrium: the inflammatory response to semen. Anim Reprod Sci (2001) 68(3-4):273–8. doi: 10.1016/S0378-4320(01)00164-6 11744271

[12] Katila T. Post-mating inflammatory responses of the uterus. Reprod Domest Anim (2012) 47(s5):31–41. doi: 10.1111/j.1439-0531.2012.02120.x 22913558

[13] Rozeboom KJ Troedsson MH.T Molitor TW Crabo BG. The effect of spermatozoa and seminal plasma on leukocyte migration into the uterus of gilts. J Anim Sci (1999) 77(8):2201–6. doi: 10.2527/1999.7782201x 10462000

[14] Marey MA Yousef MS Kowsar R Hambruch N Shimizu T Pfarrer C. Local immune system in oviduct physiology and pathophysiology: attack or tolerance/ Domest Anim Endocrinol (2016) 56 Suppl:S204–11. doi: 10.1016/j.domaniend.2016.02.005 27345318

[15] Hawk HW. Transport and fate of spermatozoa after insemination of cattle. J Dairy Sci (1987) 70(7):1487–503. doi: 10.3168/jds.S0022-0302(87)80173-X 3305615

[16] Taylor U Zerbe H Seyfert H-M Rath D Baulain U Langner KFA. Porcine spermatozoa inhibit post-breeding cytokine induction in uterine epithelial cells in vivo. Anim Reprod Sci (2009) 115(1):279–89. doi: 10.1016/j.anireprosci.2008.11.019 19136226

[17] Schjenken JE Glynn DJ Sharkey DJ Robertson SA. TLR4 signaling is a major mediator of the female tract response to seminal fluid in mice. Biol Reprod (2015) 93(3):68. doi: 10.1095/biolreprod.114.125740 26157066

[18] Schjenken JE Sharkey DJ Green ES Chan HY Matias RA Moldenhauer LM. Sperm modulate uterine immune parameters relevant to embryo implantation and reproductive success in mice. Commun Biol (2021) 4(1):572. doi: 10.1038/s42003-021-02038-9 33990675 PMC8121928

[19] Guerin LR Moldenhauer LM Prins JR Bromfield JJ Hayball JD Robertson SA. Seminal fluid regulates accumulation of FOXP3+ regulatory T cells in the preimplantation mouse uterus through expanding the FOXP3+ cell pool and CCL19-mediated Recruitment1. Biol Reprod (2011) 85(2):397–408. doi: 10.1095/biolreprod.110.088591 21389340

[20] O'Leary S Jasper M Warnes G Armstrong D Robertson S. Seminal plasma regulates endometrial cytokine expression, leukocyte recruitment and embryo development in the pig. Reproduction (2004) 128:237–47. doi: 10.1530/rep.1.00160 15280563

[21] Hamonic G Pasternak JA Ng SH Fourie KR Simko OM Deluco B. Assessment of immunological response and impacts on fertility following intrauterine vaccination delivered to swine in an artificial insemination dose. Front Immunol (2020) 11:1015. doi: 10.3389/fimmu.2020.01015 32536924 PMC7267065

[22] Choudhary P Fourie KR Ng S Hamonic G Bérubé N Popowych Y. Intrauterine immunizations trigger antigen-specific mucosal and systemic immunity in pigs and passive protection in suckling piglets. Vaccine (2021) 39(42):6322–32. doi: 10.1016/j.vaccine.2021.08.080 34535320

[23] Tokuhiro K Ikawa M Benham AM Okabe M. Protein disulfide isomerase homolog PDILT is required for quality control of sperm membrane protein ADAM3 and male fertility [corrected]. Proc Natl Acad Sci U.S.A. (2012) 109(10):3850–5. doi: 10.1073/pnas.1117963109 PMC3309714 22357757

[24] Stary G Olive A Radovic-Moreno AF Gondek D Alvarez D Basto PA. VACCINES. a mucosal vaccine against chlamydia trachomatis generates two waves of protective memory T cells. Science (2015) 348(6241):aaa8205. 26089520 10.1126/science.aaa8205 PMC4605428

[25] Gondek DC Olive AJ Stary G Starnbach MN. CD4+ T cells are necessary and sufficient to confer protection against chlamydia trachomatis infection in the murine upper genital tract. J Immunol (2012) 189(5):2441–9. doi: 10.4049/jimmunol.1103032 PMC3690950 22855710

[26] Collins MK Tay CS Erlebacher A. Dendritic cell entrapment within the pregnant uterus inhibits immune surveillance of the maternal/fetal interface in mice. J Clin Invest (2009) 119(7):2062–73. doi: 10.1172/JCI38714 PMC2701881 19546507

[27] Choudhary P Tay CS Erlebacher A. A single-dose intramuscular nanoparticle vaccine with or without prior intrauterine priming triggers specific uterine and colostral mucosal antibodies and systemic immunity in gilts but not passive protection for suckling piglets. Front Vet Sci (2022) 9:931232. doi: 10.3389/fvets.2022.931232 35990278 PMC9383261

[28] Louis L Clark M Wise MC Glennie N Wong A Broderick K. Intradermal synthetic DNA vaccination generates leishmania-specific T cells in the skin and protection against leishmania major. Infect Immun (2019) 87(8). doi: 10.1128/IAI.00227-19 PMC6652766 31182618

[29] Kim YC Jarrahian C Zehrung D Mitragotri S Prausnitz MR. Delivery systems for intradermal vaccination. Curr Top Microbiol Immunol (2012) 351:77–112. doi: 10.1007/82_2011_123 21472533 PMC3173582

[30] Mrsny RJ. Does an intradermal vaccination for monkeypox make sense? AAPS J (2022) 24(6):104. doi: 10.1208/s12248-022-00754-6 36195806 PMC9531852

[31] Tarnow K King N. Intradermal injections: traditional bevel up versus bevel down. Appl Nurs Res (2004) 17(4):275–82. doi: 10.1016/S0897-1897(04)00079-5 15573336

[32] Norman JJ Gupta J Patel SR Park S Jarrahian C Zehrung D. Reliability and accuracy of intradermal injection by mantoux technique, hypodermic needle adapter, and hollow microneedle in pigs. Drug Delivery Transl Res (2014) 4(2):126–30. doi: 10.1007/s13346-013-0184-5 25786726

[33] Lambert PH Laurent PE. Intradermal vaccine delivery: will new delivery systems transform vaccine administration? Vaccine (2008) 26(26):3197–208. doi: 10.1016/j.vaccine.2008.03.095 18486285

[34] Ochiel DO Fahey JV Ghosh M and Haddad SN Wira CR. Innate immunity in the female reproductive tract: role of sex hormones in regulating uterine epithelial cell protection against pathogens. Curr Womens Health Rev (2008) 4(2):102–17. doi: 10.2174/157340408784246395 PMC2717724 19644567

[35] Capaldo CT Nusrat A. Cytokine regulation of tight junctions. Biochim Biophys Acta (2009) 1788(4):864–71. doi: 10.1016/j.bbamem.2008.08.027 PMC2699410 18952050

[36] Fahey JV Wright JA Shen L Smith JM Ghosh M Rossoll RM. Estradiol selectively regulates innate immune function by polarized human uterine epithelial cells in culture. Mucosal Immunol (2008) 1(4):317–25. doi: 10.1038/mi.2008.20 PMC4815904 19079193

[37] Edstrom K. The porcine cervix. Uppsala: Swedish University of Agricultural Sciences (2009).

[38] Hussein AM Newby TJ Bourne FJ. Immunohistochemical studies of the local immune system in the reproductive tract of the sow. J Reprod Immunol (1983) 5(1):1–15. doi: 10.1016/0165-0378(83)90016-5 6834337

[39] Lorenzen E Follmann F Jungersen G Agerholm JS. A review of the human vs. porcine female genital tract and associated immune system in the perspective of using minipigs as a model of human genital chlamydia infection. Vet Res (2015) 46:116–6. doi: 10.1186/s13567-015-0241-9 PMC4586017 26411309

[40] Kaeoket K Persson E Dalin AM. Corrigendum to “The sow endometrium at different stages of the oestrus cycle: studies on morphological changes and infiltration by cells of the immune system”. Anim Reprod Sci (2002) 73(1):89–107. [Anim. Reprod. Sci. 65 (2001) 95–114]. doi: 10.1016/s0378-4320(00)00211-6 11182512

[41] Akira S Takeda K. Toll-like receptor signalling. Nat Rev Immunol (2004) 4(7):499–511. doi: 10.1038/nri1391 15229469

[42] Akira S Hemmi H. Recognition of pathogen-associated molecular patterns by TLR family. Immunol Lett (2003) 85(2):85–95. doi: 10.1016/S0165-2478(02)00228-6 12527213

[43] Hamonic G Pasternak JA Forsberg NM Kaser T Wilson HL. Expression of pattern recognition receptors in porcine uterine epithelial cells in vivo and in culture. Vet Immunol Immunopathol (2018) 202:1–10. doi: 10.1016/j.vetimm.2018.06.006 30078581

[44] Schaefer TM Fahey JV Wright JA Wira CR. Innate immunity in the human female reproductive tract: antiviral response of uterine epithelial cells to the TLR3 agonist Poly(I:C). J Immunol (2005) 174(2):992. doi: 10.4049/jimmunol.174.2.992 15634923

[45] Hamonic G Pasternak JA Wilson HL. Recognizing conserved non-canonical localization patterns of toll-like receptors in tissues and across species. Cell Tissue Res (2018) 372(1):1–11. doi: 10.1007/s00441-017-2767-9 29330675

[46] Pasternak JA. Intrauterine delivery of subunit vaccines induces a systemic and mucosal immune response in rabbits. Am J Reprod Immunol (2017) 78(5):e12732. doi: 10.1111/aji.12732 28771858

[47] Rozeboom KJ Troedsson MH Crabo BG. Characterization of uterine leukocyte infiltration in gilts after artificial insemination. J Reprod Fertil (1998) 114(2):195–9. doi: 10.1530/jrf.0.1140195 10070347

[48] Bischof RJ Lee CS Brandon MR Meeusen E. Inflammatory response in the pig uterus induced by seminal plasma. J Reprod Immunol (1994) 26(2):131–46. doi: 10.1016/0165-0378(94)90036-1 7932389

[49] Kaeoket K Persson E Dalin AM. The sow endometrium at different stages of the oestrous cycle: studies on morphological changes and infiltration by cells of the immune system. Anim Reprod Sci (2001) 65(1-2):95–114. doi: 10.1016/S0378-4320(00)00211-6 11182512

[50] Gączarzewicz D Udała J Piasecka M Błaszczyk B Stankiewicz T. Bacterial contamination of boar semen and its relationship to sperm quality preserved in commercial extender containing gentamicin sulfate. Polish J Vet Sci (2016) 19(3):451–9. doi: 10.1515/pjvs-2016-0057 27760038

[51] Kaeoket K Persson E Dalin AM. Corrigendum to “The sow endometrium at different stages of the oestrous cycle: studies on the distribution of CD2, CD4, CD8 and MHC class II expressing” cells. Anim Reprod Sci (2002) 73(1):109–19. [Anim. Reprod. Sci. 68 (2001) 99–109]. doi: 10.1016/s0378-4320(02)00126-4 11600278

[52] Waddell LA Lefevre L Bush SJ Raper A Young R Lisowski ZM. ADGRE1 (EMR1, F4/80) is a rapidly-evolving gene expressed in mammalian monocyte-macrophages. Front Immunol (2018) 9:2246. doi: 10.3389/fimmu.2018.02246 30327653 PMC6174849

[53] Lu L Kuroishi T Tanaka Y Furukawa M Nochi T Sugawara S. Differential expression of CD11c defines two types of tissue-resident macrophages with different origins in steady-state salivary glands. Sci Rep (2022) 12(1):931. doi: 10.1038/s41598-022-04941-5 35042931 PMC8766464

[54] Bulmer JN Williams PJ Lash GE. Immune cells in the placental bed. Int J Dev Biol (2010) 54(2-3):281–94. doi: 10.1387/ijdb.082763jb 19876837

[55] Guilliams M Ginhoux F Jakubzick C Naik SH Onai N Schraml BU. Dendritic cells, monocytes and macrophages: a unified nomenclature based on ontogeny. Nat Rev Immunol (2014) 14(8):571–8. doi: 10.1038/nri3712 PMC4638219 25033907

[56] Granot T Senda T Carpenter DJ Matsuoka N Weiner J Gordon CL. Dendritic cells display subset and tissue-specific maturation dynamics over human life. Immunity (2017) 46(3):504–15. doi: 10.1016/j.immuni.2017.02.019 PMC5415308 28329707

[57] Collin M Bigley V. Human dendritic cell subsets: an update. Immunology (2018) 154(1):3–20. doi: 10.1111/imm.12888 29313948 PMC5904714

[58] Steinman RM Inaba K Turley S Pierre P Mellman I. Antigen capture, processing, and presentation by dendritic cells: recent cell biological studies. Hum Immunol (1999) 60(7):562–7. doi: 10.1016/S0198-8859(99)00030-0 10426272

[59] Merad M Sathe P Helft J Miller J Mortha A. The dendritic cell lineage: ontogeny and function of dendritic cells and their subsets in the steady state and the inflamed setting. Annu Rev Immunol (2013) 31:563–604. doi: 10.1146/annurev-immunol-020711-074950 23516985 PMC3853342

[60] Cao Q Lu J Li Q Wang C Wang XM Lee VW. CD103+ dendritic cells elicit CD8+ T cell responses to accelerate kidney injury in adriamycin nephropathy. J Am Soc Nephrol (2016) 27(5):1344–60. doi: 10.1681/ASN.2015030229 PMC4849820 26376858

[61] Summerfield A Guzylack-Piriou L Schaub A Carrasco CP Tâche V Charley B. Porcine peripheral blood dendritic cells and natural interferon-producing cells. Immunology (2003) 110(4):440–9. doi: 10.1111/j.1365-2567.2003.01755.x PMC1783075 14632641

[62] Mayer JU Demiri M Agace WW MacDonald AS Svensson-Frej M Milling SW. Different populations of CD11b(+) dendritic cells drive Th2 responses in the small intestine and colon. Nat Commun (2017) 8:15820. doi: 10.1038/ncomms15820 28598427 PMC5472728

[63] Auray G Keller I Python S Gerber M Bruggmann R Ruggli N. Characterization and transcriptomic analysis of porcine blood conventional and plasmacytoid dendritic cells reveals striking species-specific differences. J Immunol (2016) 197(12):4791–806. doi: 10.4049/jimmunol.1600672 27837108

[64] Ashwell JD DeFranco AL Paul WE Schwartz RH. Antigen presentation by resting b cells. radiosensitivity of the antigen-presentation function and two distinct pathways of T cell activation. J Exp Med (1984) 159(3):881–905. doi: 10.1084/jem.159.3.881 6607969 PMC2187250

[65] Cruse JM Lewis RE Wang H eds. 7 - ANTIGEN PRESENTATION. In: Immunology guidebook. San Diego: Academic Press. p. 267–76.

[66] Luckheeram RV Zhou R Verma AD Xia B. CD4^+^T cells: differentiation and functions. Clin Dev Immunol 2012 (2012) p:925135. doi: 10.1155/2012/925135 PMC3312336 22474485

[67] Strutt TM McKinstry KK Swain SL. Control of innate immunity by memory CD4 T cells. Adv Exp Med Biol (2011) 780:57–68. doi: 10.1007/978-1-4419-5632-3_6 21842365 PMC3771518

[68] Prabhala RH Wira CR. Sex hormone and IL-6 regulation of antigen presentation in the female reproductive tract mucosal tissues. J Immunol (1995) 155(12):5566–73. doi: 10.4049/jimmunol.155.12.5566 7499839

[69] Wira CR Rossoll RM. Antigen-presenting cells in the female reproductive tract: influence of the estrous cycle on antigen presentation by uterine epithelial and stromal cells. Endocrinology (1995) 136(10):4526–34. doi: 10.1210/endo.136.10.7664673 7664673

[70] Brandtzaeg P Kiyono H Pabst R Russell MW. Terminology: nomenclature of mucosa-associated lymphoid tissue. Mucosal Immunol (2008) 1(1):31–7. doi: 10.1038/mi.2007.9 19079158

[71] Dalin AM Kaeoket K Persson E. Immune cell infiltration of normal and impaired sow endometrium. Anim Reprod Sci (2004) 82-83:401–13. doi: 10.1016/j.anireprosci.2004.04.012 15271469

[72] Bischof RJ Brandon MR Lee CS. Studies on the distribution of immune cells in the uteri of prepubertal and cycling gilts. J Reprod Immunol (1994) 26(2):111–29. doi: 10.1016/0165-0378(94)90035-3 7932388

[73] Kaeoket K Dalin AM Magnusson U Persson E. Corrigendum to "The sow endometrium at different stages of the oestrous cycle: studies on the distribution of CD2, CD4, CD8 and MHC class II expressing" cells. Anim Reprod Sci (2002) 73(1-2):109–19. doi: 10.1016/S0378-4320(02)00127-6 12220822

[74] Khong TY. Immunohistologic study of the leukocytic infiltrate in maternal uterine tissues in normal and preeclamptic pregnancies at term. Am J Reprod Immunol Microbiol (1987) 15(1):1–8. doi: 10.1111/j.1600-0897.1987.tb00141.x 2892429

[75] Yeaman GR Guyre PM Fanger MW Collins JE White HD Rathbun W. Unique CD8+ T cell-rich lymphoid aggregates in human uterine endometrium. J Leukoc Biol (1997) 61(4):427–35. doi: 10.1002/jlb.61.4.427 9103229

[76] Mettler L Jürgensen A Volkov NI Kulakov V Parwaresch MR. Lmmuno histochemical profile of endometrium in patients with genital endometriosis. Diagn Ther Endosc (1997) 3(3):127–45. doi: 10.1155/DTE.3.127 PMC2362564 18493428

[77] Yeaman GR Collins JE Fanger MW Wiram CR Lydyard PM. CD8+ T cells in human uterine endometrial lymphoid aggregates: evidence for accumulation of cells by trafficking. Immunology (2001) 102(4):434–40. doi: 10.1046/j.1365-2567.2001.01199.x PMC1783206 11328377

[78] Blazquez NB Batten EH Long SE Perry GC. Histology and histochemistry of the bovine reproductive tract caudal to the cervix. part i. the vestibule and associated glands. Br Vet J (1987) 143(4):328–37. doi: 10.1016/0007-1935(87)90066-2 3620891

[79] Corbeil LB Munson L Campero C BonDurant RH. Bovine trichomoniasis as a model for development of vaccines against sexually-transmitted disease. Am J Reprod Immunol (2001) 45(5):310–9. doi: 10.1111/j.8755-8920.2001.450507.x 11432406

[80] Corbeil LB Campero CM Rhyan JC Anderson ML Gershwin LJ Agnew DW. Uterine mast cells and immunoglobulin-e antibody responses during clearance of tritrichomonas foetus. Vet Pathol (2005) 42(3):282–90. doi: 10.1354/vp.42-3-282 15872374

[81] Anderson ML BonDurant RH Corbeil RR Corbeil LB. Immune and inflammatory responses to reproductive tract infection with tritrichomonas foetus in immunized and control heifers. J Parasitol (1996) 82(4):594–600. doi: 10.2307/3283783 8691366

[82] Oliveira LJ Mansourri-Attia N Fahey AG Browne J Forde N Roche JF. Correction: characterization of the Th profile of the bovine endometrium during the oestrous cycle and early pregnancy. PloS One (2014) 9(1). doi: 10.1371/annotation/93d63399-0e71-4a25-a45c-311910ee6da5 PMC5935500 29294483

[83] Oliveira LJ Mansourri-Attia N Fahey AG Browne J Forde N Roche JF. Characterization of the Th profile of the bovine endometrium during the oestrous cycle and early pregnancy. PloS One (2013) 8(10):e75571. doi: 10.1371/journal.pone.0075571 24204576 PMC3808391

[84] Bertasoli B Santos dos A Paula R Barbosa A SIlva G Jorge E. Swine placenta and placentation. Brazillian J Biol Sci (2015) 2(4):199–207.

[85] Bannunah AM Vllasaliu D Lord J Stolnik S. Mechanisms of nanoparticle internalization and transport across an intestinal epithelial cell model: effect of size and surface charge. Mol Pharmaceutics (2014) 11(12):4363–73. doi: 10.1021/mp500439c 25327847

[86] Leroy F Van Hoeck J Bogaert C. Hormonal control of pinocytosis in the uterine epithelium of the rat. J Reprod Fertility (1976) 47:59–62. doi: 10.1530/jrf.0.0470059 1271375

[87] Li Z Palaniyandi S Zeng R Tuo W Roopenian DC Zhu X. Transfer of IgG in the female genital tract by MHC class I-related neonatal fc receptor (FcRn) confers protective immunity to vaginal infection. Proc Natl Acad Sci United States America (2011) 108(11):4388–93. doi: 10.1073/pnas.1012861108 PMC3060240 21368166

[88] Richardson J Kaushic C Wira C. Polymeric immunoglobulin (Ig) receptor production and IgA transcytosis in polarized primary cultures of mature rat uterine epithelial cells. Biol Reprod (1995) 53:488–98. doi: 10.1095/biolreprod53.3.488 7578671

[89] Dickinson BL Badizadegan K Wu Z Ahouse JC Zhu X Simister NE. Bidirectional FcRn-dependent IgG transport in a polarized human intestinal epithelial cell line. J Clin Invest (1999) 104(7):903–11. doi: 10.1172/JCI6968 PMC408555 10510331

[90] Rodewald R Kraehenbuhl JP. Receptor-mediated transport of IgG. J Cell Biol (1984) 99(1 Pt 2):159s–64s. doi: 10.1083/jcb.99.1.159s PMC2275593 6235233

